# Supplementary material for: Curated and harmonised transcriptomics datasets of interstitial lung diseases
Source: Data Brief. 2025 Oct 14;63:112139. doi: 10.1016/j.dib.2025.112139 (PMC12581653; doi:10.1016/j.dib.2025.112139)
Supplement: Supplementary file 1 [file mmc1.zip › Supplementary_material/RNA-seq/fastQC reports/GSE184316/SRR15926748_fastqc.html]

SRR15926748.fastq.gz FastQC Report 

FastQC Report

Mon 5 Feb 2024  
SRR15926748.fastq.gz

## Summary

- Basic Statistics
- Per base sequence quality
- Per sequence quality scores
- Per base sequence content
- Per sequence GC content
- Per base N content
- Sequence Length Distribution
- Sequence Duplication Levels
- Overrepresented sequences
- Adapter Content

## Basic Statistics

| Measure | Value |
| --- | --- |
| Filename | SRR15926748.fastq.gz |
| File type | Conventional base calls |
| Encoding | Sanger / Illumina 1.9 |
| Total Sequences | 9468089 |
| Sequences flagged as poor quality | 0 |
| Sequence length | 25-387 |
| %GC | 49 |

## Per base sequence quality

## Per sequence quality scores

## Per base sequence content

## Per sequence GC content

## Per base N content

## Sequence Length Distribution

## Sequence Duplication Levels

## Overrepresented sequences

| Sequence | Count | Percentage | Possible Source |
| --- | --- | --- | --- |
| GGAAGACAGTGTGGCGATTCCTCAGGGATCTAGAACTAGAAATACCATTG | 151975 | 1.6051285533965725 | No Hit |
| ATTGATTCTTCCTACCCATGAGCATGGAATGTTCTTCCATTTGTTTGTAT | 99846 | 1.0545528247569282 | No Hit |
| ATAGCAGCATGATTTATAGTCCTTTGGGTATATACCCAGTAATGGGATGG | 88669 | 0.9365036598198432 | No Hit |
| GAAGGACCTCTTCAAGGAGAACTACAAACCACTGCTCAAGGAAATAAAAG | 86741 | 0.916140522126482 | No Hit |
| GAAGGACCTCTTCAAGGAGAACTACAAACCACTGCTCAATGAAATAAAAG | 58079 | 0.613418399425692 | No Hit |
| ATAGCAGCATGATTTATAATCCTTTGGGTATATACCCAGTAATGGGATGG | 43390 | 0.4582762160347247 | No Hit |
| CAGTGGGGCCACGAGCTGAGTGCGTCCTGTCACTCCACTCCCATGTCCCT | 35651 | 0.3765384968392249 | No Hit |
| CACCTCCCCGAAGCTCAGGGAGAGCCCTGTTAGGGCCGCCTCTGGCCCTA | 34322 | 0.36250187339810597 | No Hit |
| AGACATCAAATCCTGCAGACAAGGGGAGCCCTCAGTCTGCAGGGCTCCAT | 28386 | 0.29980706771979015 | No Hit |
| GGCATCAAAAGTGATGGACTGCCCATTGCTGGAGAAGACCTTCTCTCCTA | 28131 | 0.2971138104003881 | No Hit |
| ATTGATTCTTCCTACCCATGAGCATGGAATGTTCTTCCATTTGTTTGTGT | 24703 | 0.26090798259289705 | No Hit |
| GGAAGACAGTGTGGCGATTCCTCAAGGATCTAGAACTAGAAATACCATTG | 24050 | 0.2540111314965459 | No Hit |
| AGGCTACACACTGAGGACTCTGTTCCTCCCCTTTCCGCCTAGGGGAAAGT | 21524 | 0.22733204134435153 | No Hit |
| GCATGTGTCTTTATAGCAGCATGATTTATAGTCCTTTGGGTATATACCCA | 20997 | 0.2217659762175873 | No Hit |
| GGCCATTTTCATGATATTGATTCTTCCTACCCATGAGCATGGAATGTTCT | 19743 | 0.2085214872821749 | No Hit |
| GGAAGACAGTGTGGTGATTCCTCAGGGATCTAGAACTAGAAATACCATTG | 19439 | 0.20531070208571126 | No Hit |
| TGATATGGTTCCTGGCAAGCCCATGTGTGTTGAGAGCTTCTCAGACTATC | 19051 | 0.2012127262428564 | No Hit |
| GGAAGACAGTGTGGCGATTCCTCAGGGATCTAGAACTGGAAATACCATTG | 17592 | 0.18580306965851293 | No Hit |
| ATAGCAGCATGATTTATAGTCATTTGGGTATATACCCAGTAATGGGATGG | 17193 | 0.18158891408815442 | No Hit |
| GGGAAGCGGGGAATGTCTACGTGCGTATGCACGTGGCACTCTCTGCCCGA | 17124 | 0.18086015034290448 | No Hit |
| TGATGACACCCACCGCAACTGTCTGTCTCATATCACGAACAGCAAAGCGA | 16464 | 0.1738893666926874 | No Hit |
| CTTCATGATGTAGCAGCAGGTGCCAGGGGCTGGCTTGTAGGCGATCAGCA | 16434 | 0.17357251289040482 | No Hit |
| GGCAATGAGCGGTTCCGCTGCCCTGAGGCACTCTTCCAGCCTTCCTTCCT | 15970 | 0.16867184074843403 | No Hit |
| GGAAGACAGTGTGGCGATTCCTCAGGGATCTAGAACTAGAAATACCATTT | 15958 | 0.168545099227521 | No Hit |
| GGAAGACAGTGTGGCAATTCCTCAGGGATCTAGAACTAGAAATACCATTG | 15783 | 0.16669678538087251 | No Hit |
| TACCACTGCCACCTTCTCCATCGGCTCCACTGGCCTCGTGGTGTATGACT | 15012 | 0.15855364266220986 | No Hit |
| GTCCACGTCACACTTCATGATGGAGTTGAAGGTAGTTTCGTGGATGCCAC | 13766 | 0.14539364807407282 | No Hit |
| ATTGATTCTTCCTATCCATGAGCATGGAATGTTCTTCCATTTGTTTGTGT | 13084 | 0.13819050496884852 | No Hit |
| TTACAAAAGCAGAATAAAAGCGAAAAGAAATGAAAATGTTACACTACATT | 12458 | 0.13157882229455173 | No Hit |
| GGAAGACAGTGTGGTGATTCCTCAAGGATCTAGAACTAGAAATACCATTG | 12408 | 0.13105073262408073 | No Hit |
| GCATGTGTCTTTATAGCAGCATGATTTATAATCCTTTGGGTATATACCCA | 12330 | 0.130226912738146 | No Hit |
| CAATCCACTTGATCCCAACTCATCTCTCATTTATTTCGGCTTCTTTTATT | 12226 | 0.12912848622356635 | No Hit |
| GAAGGACCTCTTCAAGGAGAACTACAAACCACTGCTCAACGAAATAAAAG | 11489 | 0.12134444448082396 | No Hit |
| GGAAGACAGTGTGGCAATTCCTCAAGGATCTAGAACTAGAAATACCATTG | 11022 | 0.11641208695862491 | No Hit |
| ATTGATTCTTCCTACCCATGAGCATGGAATGTTCTTCCATTTCTTTGTAT | 9673 | 0.10216422764931761 | No Hit |

## Adapter Content

Produced by FastQC (version 0.11.7)
